# Gelatine-Coated Carbonyl Iron Particles and Their Utilization in Magnetorheological Suspensions

**DOI:** 10.3390/ma14102503

**Published:** 2021-05-12

**Authors:** Tomas Plachy, Patrik Rohrer, Pavlina Holcapkova

**Affiliations:** Centre of Polymer Systems, University Institute, Tomas Bata University in Zlín, třída Tomáše Bati 5678, 760 01 Zlín, Czech Republic; Patrik.rohrer@icloud.com (P.R.); holcapkova@utb.cz (P.H.)

**Keywords:** magnetorheology, gelatine, Robertson–Stiff model, carbonyl iron, core–shell

## Abstract

This study demonstrates the formation of biocompatible magnetic particles into organized structures upon the application of an external magnetic field. The capability to create the structures was examined in silicone-oil suspensions and in a gelatine solution, which is commonly used as a blood plasma expander. Firstly, the carbonyl iron particles were successfully coated with gelatine, mixed with a liquid medium in order to form a magnetorheological suspension, and subsequently the possibility of controlling their rheological parameters via a magnetic field was observed using a rotational rheometer with an external magnetic cell. Scanning electron microscopy, infrared spectroscopy, and thermogravimetric analysis confirmed the successful coating process. The prepared magnetorheological suspensions exhibited a transition from pseudoplastic to Bingham behavior, which confirms their capability to create chain-like structures upon application of a magnetic field, which thus prevents the liquid medium from flowing. The observed dynamic yield stresses were calculated using Robertson–Stiff model, which fit the flow curves of the prepared magnetorheological suspensions well.

## 1. Introduction

Magnetic particles have found use in many miscellaneous applications among various industry fields. From a human point of view, one of the most important areas is their use in biomedicine. The magnetic particles are here used especially as contrast agents for magnetic resonance imaging [1] or as agents for the magnetic separation of cells or other matters [2]; however, many other applications have been proposed for them: as carriers for control drug delivery systems [2], as a system for controlled release, as particles for hyperthermia [3,4], for embolization of the blood vessel [5,6], or their combination in arterial embolization hyperthermia [7]. Last but not least, their introduction into hydrogels can lead to the creation of artificial muscles [8]. Thus, magnetic particles can be applied as both therapeutic and diagnostic tools. For the use of magnetic particles in vivo in the biomedical field, the particles have to be biocompatible and harmless for the human body. In some of the above-mentioned applications, it is also desirable to attach proper molecules responsible for targeting the particles in the desired places, to attach proper ligands, or to maintain certain dose of drugs. These demands can be achieved through coating of the magnetic particles, which leads to the creation of core/shell structures. Many materials, such as polysaccharides [9,10], silica [7], polylactide, etc. [11,12], have been used as shells on magnetic particles in order to increase their biocompatibility. Gelatine has also been intensively investigated as a shell material for the coating of magnetic particles [13,14,15,16] in order to provide a biocompatible shell that can be further functionalized with bioactive substances. Such materials can be further used for in vivo applications such as magnetic separation, controlled drug delivery, embolization of a blood vessel, or arterial embolization hyperthermia, etc.

Arterial embolization hyperthermia is a special technique among them, since, unlike the other techniques where the particles act individually, the magnetic interaction between the particles is a crucial phenomenon for effective embolization. This creation of chain-like structures upon the application of an external magnetic field responsible for successful embolization of the blood vessel is similar to the phenomenon exhibited by magnetorheological (MR) fluids.

Magnetorheological suspensions are suspensions consisting of magnetic particles dispersed in a non-magnetic liquid medium [17,18,19]. The viscosity of such suspensions can then be controlled by an external magnetic field considering the capability of the magnetic particles to join themselves into chain-like structures, preventing the liquid medium from flowing in the perpendicular direction to the applied magnetic field [20,21]. Many MR suspensions in which mainly carbonyl iron (CI) or ferrite oxides particles are used in the mixture with silicone oil have been presented.

This study concerns a new method of coating commercial CI magnetic particles with biocompatible gelatine, which has previously been introduced to the surface of CI particles as a grafting agent to further modify the surface with graphite oxide [13] or carbon nanotubes [22]. In this study, composite particles CI/gelatine were used as the dispersed phase for a MR suspension, and their ability to form stiff structures within the liquid medium, simulating in vivo conditions, was examined. 

## 2. Experimental

### 2.1. Chemicals

Carbonyl iron particles (ER grade, *d*_50_ = 4.5 μm; the content of iron particles > 97%) were obtained from BASF Company (Ludwigshafen, Germany). Concentrated hydrochloric acid (HCl; 35%) and acetic anhydride (p.a.) were produced by Penta (Prague, Czech Republic); (3-aminopropyl)triethoxysilane (APTES, purity 98%) and gelatine type B containing more than 70% of proteins were obtained from Sigma Aldrich (St. Louis, MO, USA). Dichlormethane (p.a.) was supplied by Lachema (Brno, Czech Republic). All these substances were used as delivered without any purification. Pyridine was purified before its use by distillation on molecular sieves. Common chemicals like ethanol, toluene, and acetone (all of the grade p.a.) were supplied by Penta (Prague, Czech Republic) and used as obtained.

### 2.2. Coating of the Carbonyl Iron Particles

Carbonyl iron particles were firstly treated in 0.5 M HCl under vigorous stirring with an overhead stirrer (EUROSTAR digital IKA-Werke, Breisgau, Germany) in order to remove the low-magnetic surface layer consisting of iron oxides and other impurities from the particles, and to activate their surface [23]. The particles were then rinsed with distilled water, ethanol, and acetone, and dried in a vacuum oven (MEMMERT VO400, Memmert GmbH + Co.KG, Büchenbach, Germany) at 40 °C. Such particles were used as a reference material and are hereafter labelled as CI ER particles.

**Figure 1:**
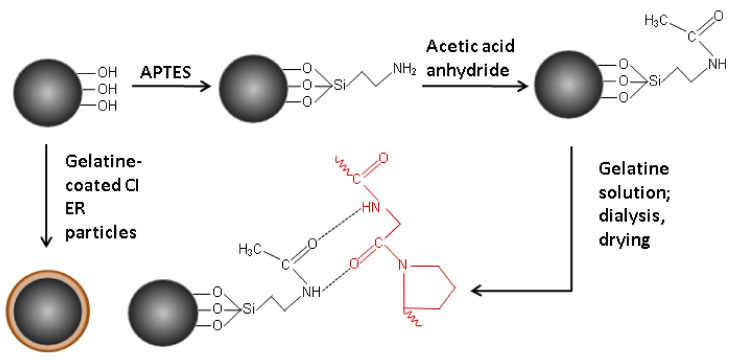
The scheme of the coating of CI particles with gelatine.

Another batch of the particles was prepared by modification of the surface of CI ER particles and their subsequent coating with gelatine (Figure 1). Firstly, the surface of the particles was modified by APTES [19,23,24] by treating of the particles in a solution consisting of a high excess of toluene and 10 mL of APTES for 6 h at 110 °C. After 6 h, particles were removed; rinsed with toluene, ethanol, and acetone; and dried at 60 °C in a vacuum oven. Three grams of the particles were then immersed in a mixture of 2 mL toluene, 0.5 mL pyridine, and 0.5 mL acetic anhydride for 16 h under vigorous stirring. In this step, amine groups were exchanged by amide groups [25] consisting of oxygen atoms, which help to create stronger hydrogen bonds that are, together with electrostatic forces, responsible for entrapping gelatine on the surface of CI particles. After 16 h, particles were carefully washed with toluene, dichloromethane, and distilled water. The particles were further treated in a 6% gelatine solution (*w/w*) for 16 h at 37 °C under vigorous stirring with the overhead stirrer in order to introduce the gelatine on the surface of the particles. Particles were then carefully rinsed with distilled water and transferred into a dialysis membrane (dialysis tubing cellulose membrane; typical molecular weight cut-off 14,000). They were dialyzed at 37 °C for 24 h in an excess of demineralized water (*σ* < 1 µS cm^−1^) in order to properly rinse the particles and remove the remaining gelatine according to the literature. The presence of excluded gelatine in the demineralized water was followed by a precipitation reaction of gelatine with a tanning agent. Particles were then washed again with the distilled water and dried in a vacuum oven at a low pressure. The so-prepared particles are hereafter labelled as CI ER-gelatine.

### 2.3. Characterization of the Particles

A scanning electron microscope (SEM; VEGA II LMU, Tescan, Brno, Czech Republic) was used to determine morphology and dimensions of the particles, and to confirm the presence of gelatine on the surface of CI ER-gelatine particles. The thermal stability of the particles within the temperature range of 25–600 °C was investigated by a thermogravimetric analysis using a thermogravimetric analyzer (TGA; TA Q500, TA Instruments, New Castle, DE, USA). The test ran under a nitrogen atmosphere at a heating rate 3 K min^−1^. Fourier transform infrared spectroscopy was used to confirm the presence of gelatine on the surface of the CI particles. The FTIR (Fourier Transform Infrared Spectroscopy) spectra were obtained in the mid-infrared region via the attenuated total reflection (ATR) technique using a diamond crystal (FT-IR spectrometer Nicolet 6700, Thermo Scientific, Waltham, MA, USA). The magnetization hysteresis curves of the particles were obtained by a vibrational sample magnetometer (EG&G PARC 704, Lake Shore, MD, USA) in magnetic fields up to 760 kA×m^−1^.

### 2.4. Preparation of MR Suspensions

Magnetorheological suspensions containing 60 wt % of the magnetic particles were prepared by mixing the prepared particles and 6% gelatine solution at a ratio of 6:4 (*w*/*w*) or by mixing them with silicone oil (Lukosiol M200, Lučební závody Kolín, Czech Republic, viscosity η = 194 mPa s, conductivity σ ≈ 10−11 S cm^−1^) in the same ratio (6:4 (*w/w*)). The gelatine solution at this concentration is widely used as a blood expander [26,27]; therefore, it was chosen in this study as a suitable liquid medium for simulating conditions of human blood from the rheological point of view. Before each measurement, the MR suspensions were manually mixed for 5 min with a glass stick to ensure the homogenous distribution of the particles within the suspension.

### 2.5. Rheological Measurements

Rheological measurements were carried out using a rotational rheometer (Physica MCR 502, Anton Paar, Graz, Austria) with a parallel plate geometry (a gap of 0.3 mm) and an external source of a magnetic field. The influence of the magnetic field, which was applied in the perpendicular direction to the flow between the parallel plates, on the rheological behavior of the prepared MR suspensions was studied within the magnetic field strengths of 87–438 kA×m^−1^. The magnetic field strengths were measured using a FH 51 Gauss teslameter (MAGNET-PHYSIK, Cologne, Germany) in the empty magnetic cell. Before the measurement in the presence of a magnetic field, the magnetic field was applied 1 min before the measurement to provide enough time for the particles to create chain-like structures within the MR suspensions. The measurements in the absence as well as in the presence of a magnetic field were carried out at shear rates 0.1–200 s^−1^ at 23 °C, where the temperature was controlled using an external thermoregulator (Julabo FS 18; JULABO GmbH, Seelbach, Germany); after each measurement, the created internal structures of the particles were destroyed by an applied shear rate of 40 s^−1^. The measurements were performed 3 times with a fresh sample for each MR suspension.

### 2.6. Sedimentation Test

The sedimentation stability of the prepared MR suspensions was evaluated using a Tensiometer Krüss K100 (MK2/SF/C, GmbH, Hamburg, Germany) with a SH0640 measuring probe. The sensor records the weight gain of the particles in the measuring probe over time and, from the dependence, the stability of the suspension can be evaluated. For the sedimentation test, diluted MR suspensions containing only 10 wt % of the particles were used.

### 2.7. Optical Microscopy

The MR suspensions prepared with 20 wt % dispersed in the silicone oil were investigated using optical microscopy (LEICA DV M2500 optical microscope, Leica Microsystems, Wetzlar, Germany) to observe the formation of magnetic-field-induced structures. The samples were priori homogenized, then put between two glasses; the chain-like structures formed under a magnetic field with a magnetic flux density of 120 mT.

## 3. Results and Discussion

### Particles Characterization

**Figure 2:**
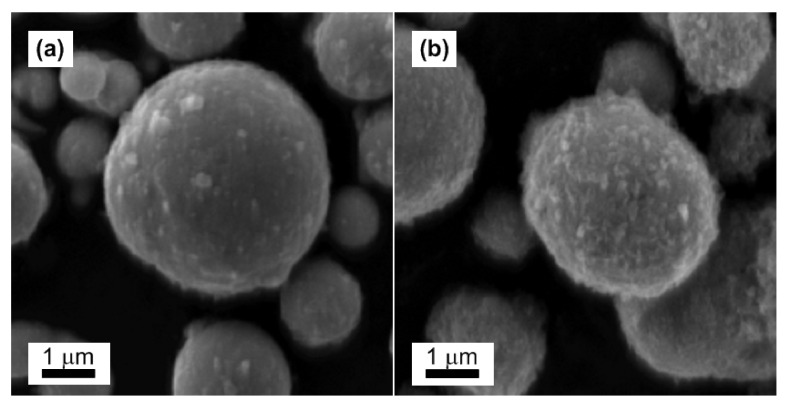
SEM images of CI ER particles (**a**) and CI ER-gelatine particles (**b**).

The original CI ER particles possessed spherical shapes and smooth surfaces (Figure 2a). The rougher surface of the CI ER-gelatine particles (Figure 2b) confirmed the successful coating of CI ER particles with gelatine, and the finding that they preserved the spherical shape represented a uniform and complete coating. The coating process did not considerably affect the size of the particles, confirming the presence of a thin layer on the surface.

**Figure 3:**
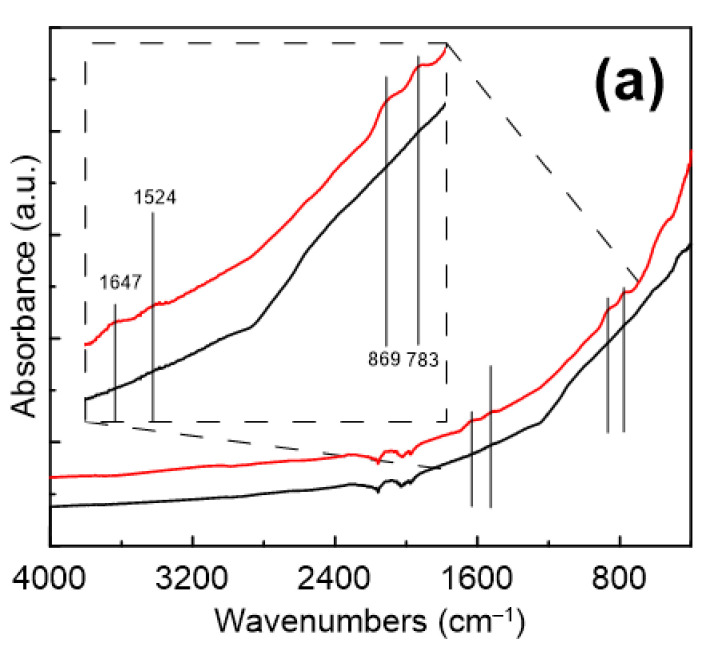

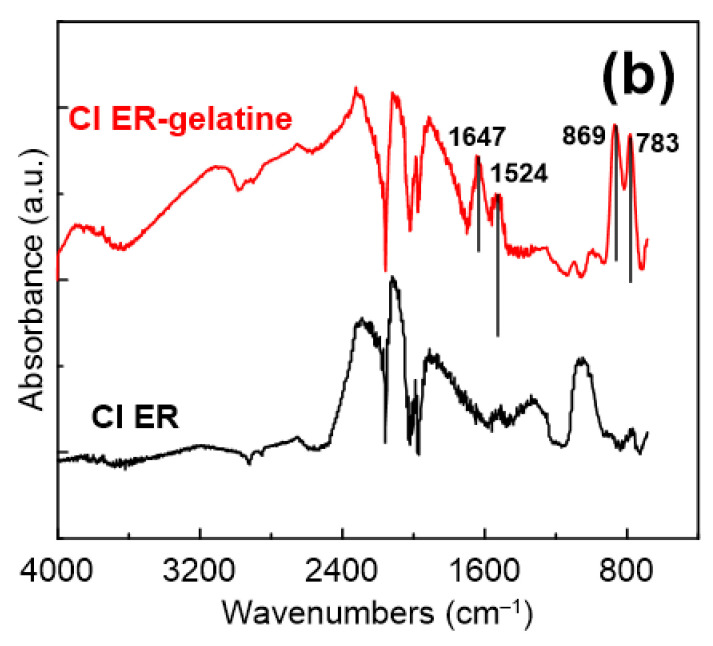
FTIR spectra of CI ER particles (black line) and CI ER-gelatine (red line) particles. (**a**) The inset picture represents a zoom of an area between approximately 1700 and 650 cm^−1^. (**b**) FTIR spectra after the baseline correction.

Infrared spectroscopy was used to confirm the presence of a gelatine coating on the surface CI ER particles. Even though the obtained spectra were strongly suppressed due to low thickness of the gelatine layer and its electrostatic attraction to the surface, changes representing the presence of gelatine were found in the spectra (Figure 3a). While the CI particles do not exhibit any characteristic peaks in the measured region, suppressed peaks determining the presence of gelatine in the spectrum of the CI ER-gelatine particles can be found in the inset. The inset shows the peaks at 1647 and 1524 cm^−1^, which represent C = O and –NH– bending [13,28,29], respectively. Furthermore, the peak at 869 cm^−1^ denotes –C-–N– group and the one at 783 cm^−1^ represents the NH group from the –CONH– cis conformation, which are typical groups observed for gelatine. Figure 3b shows the modified spectra after baseline correction, where the above-mentioned peaks are more prominent.

**Figure 4:**
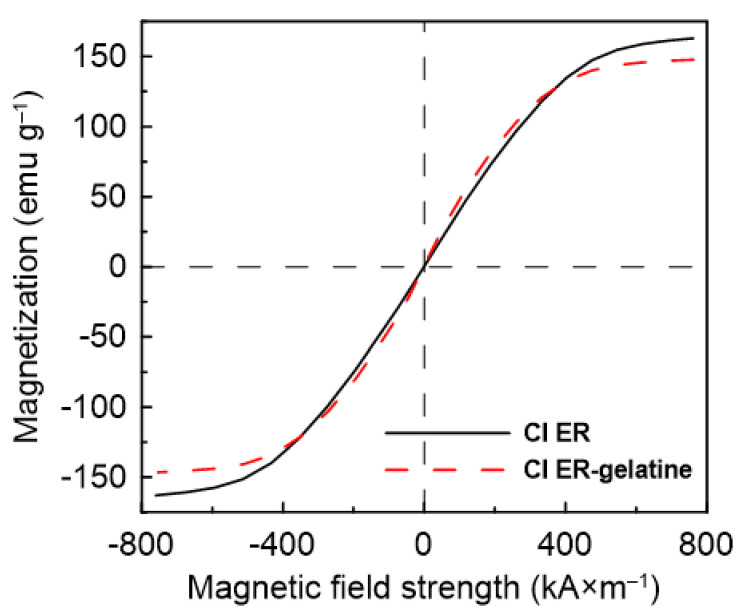
Magnetization curves for CI ER particles (black solid line) and CI ER-gelatine particles (red dashed dot line).

Magnetic properties of the particles can be determined from Figure 4. The CI ER particles exhibit higher values of magnetic saturation (*M*_S_ = 162.99 emu/g) than the CI ER-gelatine particles (*M*_S_ = 147.12 emu/g), predicting the higher MR effect for the suspensions based on the CI ER particles. The slight decrease in the value of saturation magnetization for CI ER-gelatine particles is a consequence of the non-magnetic gelatine present on their surface. Nevertheless, when compared with some studies published before, the decrease is only decent (less than 10%). Thus, coating with gelatine not only introduced a biocompatible layer on the surface of CI particles, but also obtained magnetic particles with sufficient *M*_s_ [30,31].

**Figure 5:**
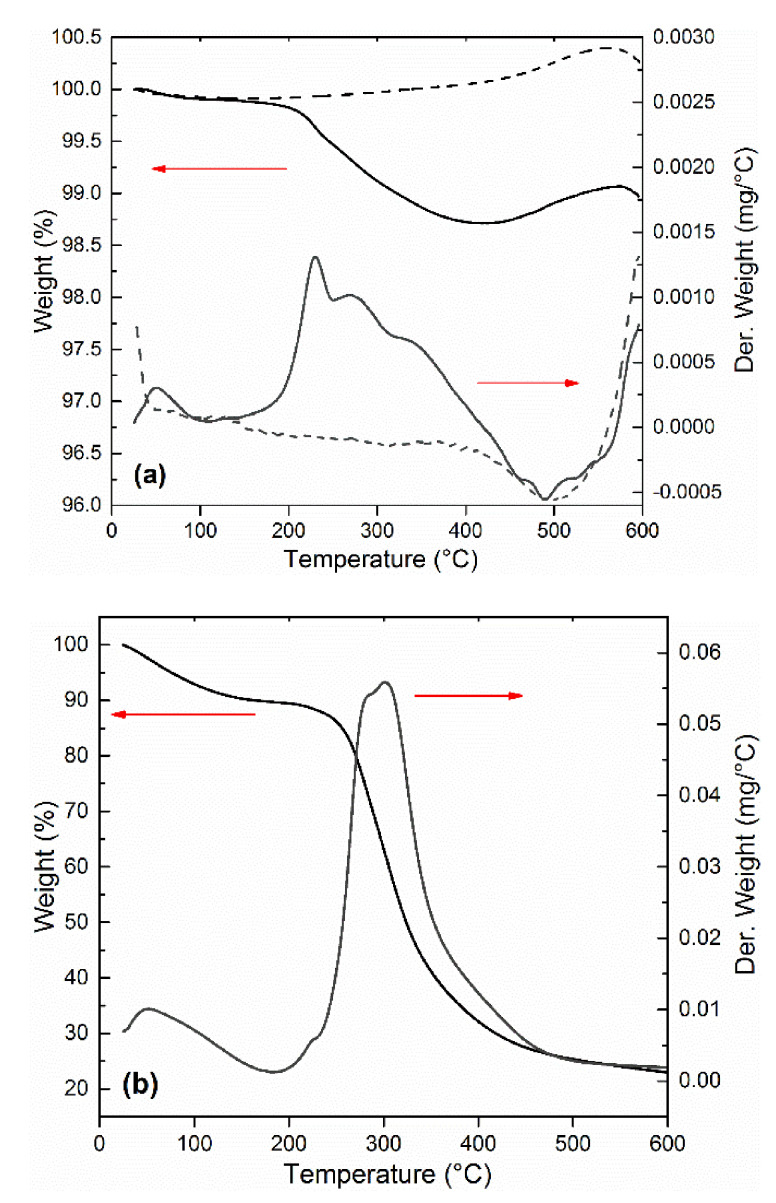
Thermogravimetric analysis of (**a**) CI ER (dashed line) and CI ER-gelatine (solid line) and (**b**) pure gelatine.

Figure 5a depicts the thermal stability of the pure CI ER and CI ER-gelatine particles. The thermal decomposition for pure gelatine is shown in Figure 5b for comparison with its decomposition temperature. TGA further served as an indirect method to compare the changes between coated and uncoated particles and thus to confirm the presence of an organic layer on the surface of the CI particles. Pure CI ER and CI ER-gelatine particles exhibited a weight loss up to 200 °C due to evaporating of the adsorbed moisture. In the case of pure gelatine (Figure 5b), this loss can be observed at same temperature region, but it is much more prominent due to higher amount of water adsorbed on the surface of gelatine. The second weight loss above 200 °C is attributed to protein thermal degradation or decomposition. In the case of pure gelatine (Figure 5b), this process proceeds in one step up to 500 °C. For the pure CI ER, no weight loss between 200 and 500 °C is observed; conversely, the slight increase up to 550 °C representing the formation of oxides on the surface of carbonyl iron due to remnant amounts of omnipresent oxygen present on the particles or the formation of various nitrides such as Fe_4_N or Fe_3_N_x_ cannot be excluded [32,33]. In the case of CI ER-gelatine particles in the temperature region 200–500 °C (Figure 5a), processes of degradation or decomposition of gelatine and the formation of various oxides on the surface of the particles occur at the same time. This can explain the more complicated curve of derivative weight representing more processes compared with a pure gelatine curve. Over 500 °C, most gelatine decomposes, the process of formation of various oxides on the iron surface starts to dominate, and the shape of the curves of CI ER-gelatine particles copies the curves of CI ER particles. In addition, the low amount of gelatine (less than 2%) fits well with the high *M*_s_ of CI ER-gelatine particles.

**Figure 6:**
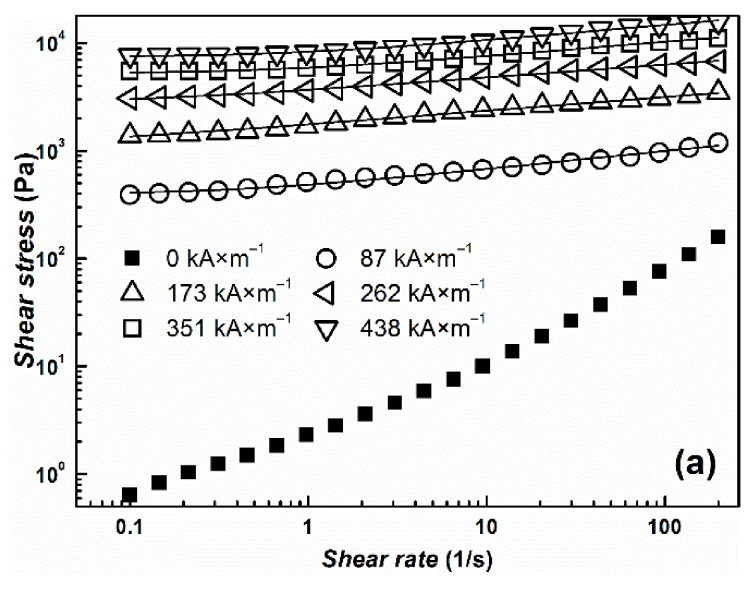

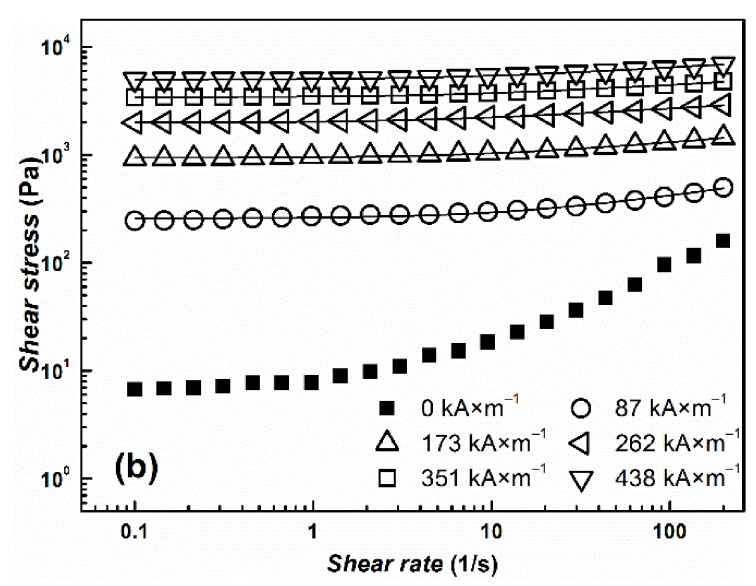
The log-log dependence of shear stress, *τ*, on the shear rate, $\dot{\gamma }$, for prepared silicone–oil suspensions of (**a**) bare CI particles (**b**) and/or CI ER-gelatine particles. The solid lines represent the fit from the R–S model.

**Figure 7:**
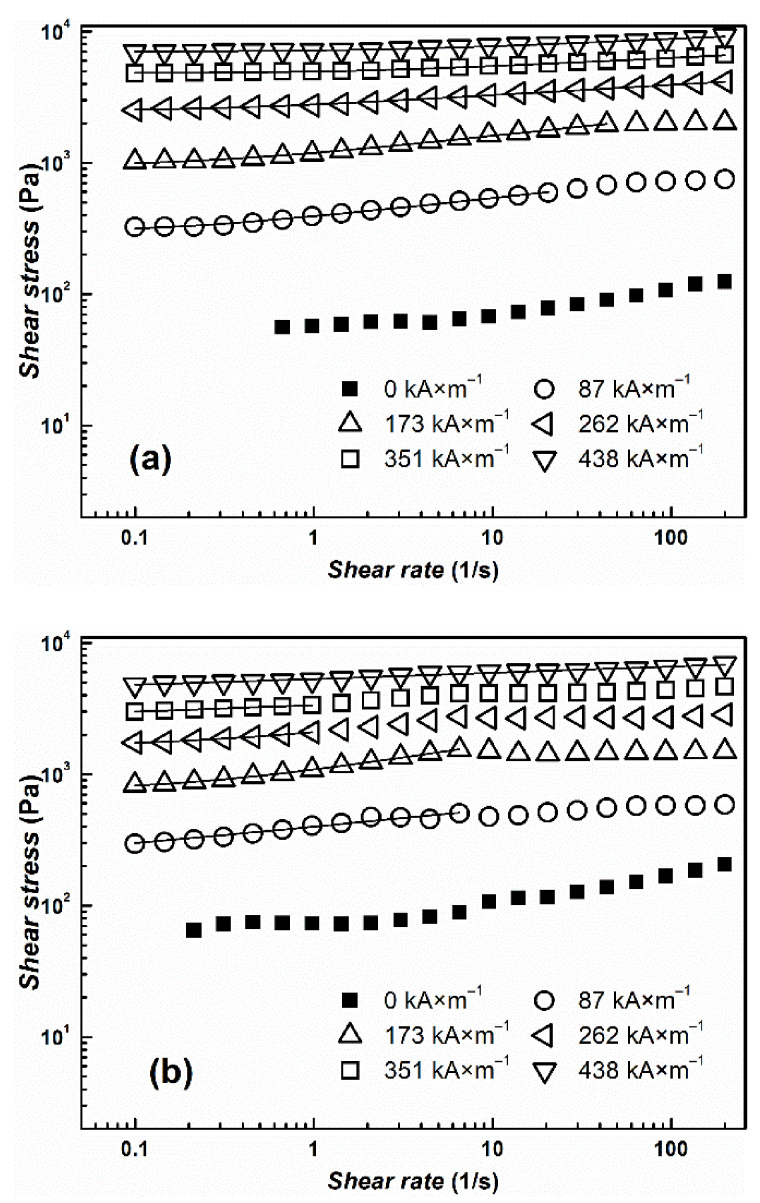
The log-log dependence of shear stress, *τ*, on the shear rate, $\dot{\gamma }$, for prepared MR suspensions of (**a**) bare CI particles (**b**) and/or CI ER-gelatine particles. As a liquid medium, a 6% gelatine solution was used. The solid lines represent the fit from the R–S model. Only one curve for each sample from 3 measurements was used as a representative and is shown in the figure.

The transformation from a liquid-like to a solid-like state is important for the potential use of the suspensions for embolization or arterial embolization hyperthermia. This effect can be easily observed by measuring their rheological parameters in the absence and in the presence of an external magnetic field. Both prepared MR suspensions undergo the dramatic change in viscosity from pseudoplastic behavior into Bingham fluids, which are able to withstand certain stress before they start to flow (Figure 6). The more prominent pseudoplastic behavior of CI ER-gelatine particles can be attributed to an increased amount of interactions between silicone oil and particles due to the presence of gelatine. The further increase in rheological parameters with increasing magnetic field confirms the formation of stiff structures within the suspension, preventing the fluid from flowing. Although the CI ER-gelatine particles exhibited lower *M*_S_, the decrease in the MR effect of their MR suspensions was negligible in comparison with the CI ER-based MR suspension. Figure 7 shows the behavior of prepared particles mixed with a 6% gelatine solution instead of silicone oil. The observed yield stresses are lower than in the case of MR suspensions based on silicone oil (Table 1). The gelatine solution contains polymer chains, which hinder the close packaging of magnetic particles into the chains, prolonging the distance between them, and thus lowering the overall magnetic force acting in the created chains. Thus, even though the particles are able to create chain-like structures in this medium, the presence of matter that can act as a steric barrier can lower the MR performance of the prepared MR suspensions. Generally, the magnetic-field-induced yield stress of MR suspensions is their important feature from an application point of view and the Robertson–Stiff (R–S) model (Equation (1)), which has been recently successfully used for modeling of flow curves for MR suspension [34,35], was implemented to obtain exact *τ*_y_ values. In the R–S model, *K* and *n* represent consistency and flow behavior index, respectively.

(1) $$\tau ={\left[K^{\frac{1}{n}}{\left|\overset{\cdot }{\gamma }\right|}^{\frac{n-1}{n}}+{\left(\frac{\tau_{y}}{\left|\overset{\cdot }{\gamma }\right|}\right)}^{\frac{1}{n}}\right]}^{n}\overset{\cdot }{\gamma }$$

From Figure 6 and Figure 7, it can be seen that the model fits the MR curve of the prepared systems well and enables the estimation of *τ*_y_ values. As mentioned above, use of a 6% gelatine solution instead of silicone oil led to a decrease in *τ*_y_ values due to an increase in the distance between magnetic particles. It should be also noted that even though values of *M*_S_ decreased only about 10% for CI ER-gelatine particles, the decrease in *τ*_y_ values was even more than 30% at high magnetic fields, confirming that, at high magnetic fields, *τ*_y_ scales as *τ*_y_~*M*_S_^2^ [36], producing values demonstrating saturation magnetization’s dominant impact. It should be also noted that the R–S fits for the systems in a 6% gelatine solution were performed only from the first part of the flow curves (Figure 7) since, at higher shear rates, the systems underwent significant disturbances, probably due to either a polymer chain present in the solution or their tendencies to create gelatinous structures, which can influence the fitting parameters [37].

**Figure 8:**
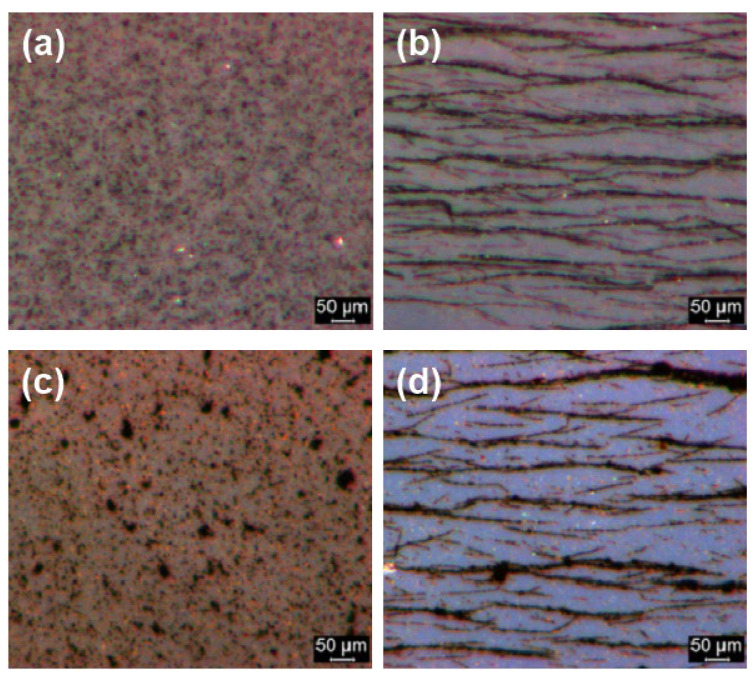
Optical microscopy of diluted silicone oil suspensions (20 wt %) in the absence (**a**,**c**) and presence (**b**,**d**) of an external magnetic field with a magnetic flux density of 120 mT for MR suspensions based on (**a**,**b**) CI ER particles and on (**c**,**d**) CI-ER gelatine particles. The direction of the applied magnetic field is from left to right.

The results of optical microscopy showed that while CI ER particles were well-dispersed in the silicone oil (Figure 8a), CI ER-gelatine particles tended to create small agglomerates, possibly due to their higher polarity and interactions between the gelatine functional group (Figure 8c). Nevertheless, both of the particles were randomly distributed within the silicone oil in the absence of a magnetic field. Upon its application, however, both materials created magnetic-field-induced structures in the direction of the applied magnetic field (Figure 8b,d). This formation proposes these materials are suitable candidates for use in various embolization techniques. It can be seen that the induced structures of CI ER particles are slightly thicker, probably due to agglomeration of the particles.

**Figure 9:**
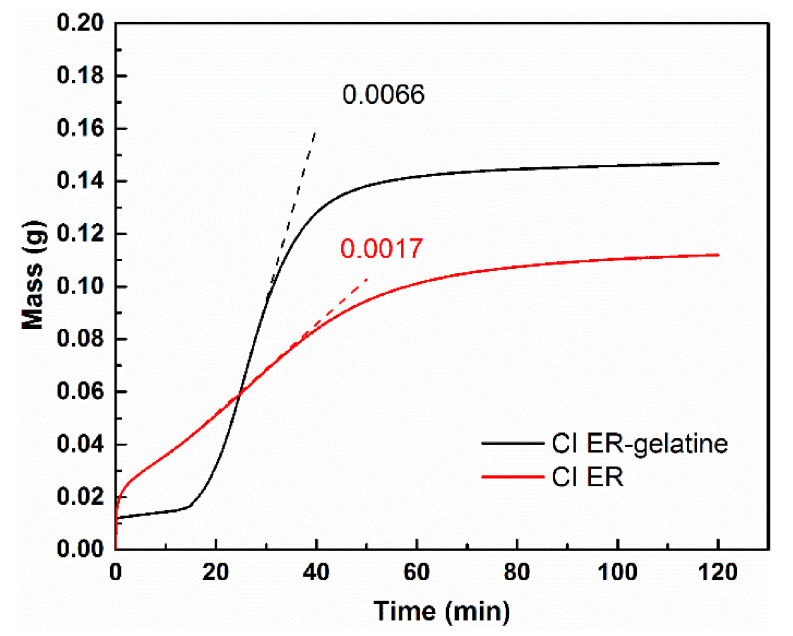
The sedimentation measurement of the prepared MR suspensions expressed as a dependence of mass increase in the particles in the measuring probe on time. The numbers in the figure represent slope of the curves associated with the highest increase in the mass.

The low sedimentation stability of suspensions can lead to their dramatic change in functional properties and, together with low redispersibility, can significantly shorten their service-life; thus, sedimentation stability plays an important role in their applicability. In this study, the sedimentation stability was evaluated according to the slope of the curve in the area with the highest increase in the particles in the measuring probe, and the time from the start of the measurement needed for 90% of the particles to settle was calculated. In the former, the MR suspension based on coated CI ER-gelatine particles demonstrated a slope of the curve of 0.0017, which is more than four times lower than that for the MR suspensions based on the bare particles. In addition, the time needed to settle 90% of the particles was found to be 59.3 and 42.8 min, respectively (Figure 9). These results are consistent with the findings in the recent literature [24,33]. The coating of the CI particles thus led to higher sedimentation stability due to several possible mechanisms: (i) lower overall density of the core–shell particles, (ii) higher friction between the particles and the liquid medium, and (iii) better compatibility of the coated particles with the liquid medium suppressing agglomeration of the particles, etc. Nevertheless, the coating process of the particles did not fully suppress the sedimentation process. In the case of the MR suspensions, this has to be achieved through the use of thickening agents or other additives [38], whose effects can be emphasized using coated particles. The uneven start of the particles can possibly arise from different characteristics of the particles affecting the interaction between the sedimentation probe and the liquid itself; nevertheless, it should not further affect the sedimentation process.

## 4. Conclusions

Gelatine-coated magnetic particles were prepared via a coating process of commercial carbonyl iron particles with gelatine. The presence of gelatine on the surface of the particles was confirmed by scanning electron microscopy and infrared spectroscopy. The prepared particles were mixed with a 6% gelatine solution or silicone oil in order to create magnetorheological suspensions. The capability of the particles to create organized structures within the gelatine solution under the application of a magnetic field was further confirmed using magnetorheological measurements. The suspension exhibited an abrupt increase in shear stress, which suggests their possible utility, for example, in embolization processes. The Robertson–Stiff model was used to estimate the yield stress values of prepared the magnetorheological suspensions when we found that using the gelatine solution as a liquid carrier led to a decrease in yield stress values due to the presence of polymer chains acting as steric barriers increasing the distance between magnetic particles. The prepared magnetic particles, creating aligned structures in the direction of an applied magnetic field exhibiting yield stress, are suitable candidates for use in embolization of blood vessels or in arterial embolization hyperthermia, where the gelatine present can additionally serve as a functional site for other suitable substances.

## Figures and Tables

**Table 1:** Values of yield stress determined from the R–S model for the prepared MR suspensions. The average values were calculated from 3 measurements performed with a fresh sample.

	Yield Stress (Pa)			
Magnetic Field Intensity (kA×m^−1^)	Silicone-Oil		6% Gelatine Solution	
	CI ER	CI ER-Gelatine	CI ER	CI ER-Gelatine
87	370.0 ± 30	270.0 ± 15	300.0 ± 10	220.0 ± 10
173	1100 ± 60	940.0 ± 10	970.0 ± 20	800.0 ± 80
262	2800 ± 50	2000 ± 30	2600 ± 100	1700 ± 90
351	5100 ± 70	3500 ± 20	4900 ± 100	2800 ± 70
438	7400 ± 80	5000 ± 10	7200 ± 100	4400 ± 80

MR—magnetorheological; CI ER—carbonyl iron particles, grade ER.

## Data Availability

The data presented in this study are available on request from the corresponding author.

## References

[1] Srinivas M., Boehm-Sturm P., Figdor C.G., de Vries I.J., Hoehn M. (2012). Labeling cells for in vivo tracking using F-19 MRI. Biomaterials.

[2] Pankhurst Q.A., Connolly J., Jones S.K., Dobson J. (2003). Applications of magnetic nanoparticles in biomedicine. J. Phys. D Appl. Phys..

[3] Kashevsky B.E., Kashevsky S.B., Korenkov V.S., Istomin Y.P., Terpinskaya T.I., Ulashchik V.S. (2015). Magnetic hyperthermia with hard-magnetic nanoparticles. J. Magn. Magn. Mater..

[4] Zhao L.Y., Liu J.Y., Ouyang W.W., Li D.Y., Li L., Li L.Y., Tang J.T. (2013). Magnetic-mediated hyperthermia for cancer treatment: Research progress and clinical trials. Chin. Phys. B.

[5] Iacob G., Ciochina A.D., Bredetean O., Racuciu M. (2008). Magnetite particle utilization for blood vessel embolization—A practical modeling. Optoelectron. Adv. Mater. Rapid Commun..

[6] Michaud F., Li N., Plantefeve R., Nosrati Z., Tremblay C., Saatchi K., Moran G., Bigot A., Hafeli U.O., Kadoury S. (2019). Selective embolization with magnetized microbeads using magnetic resonance navigation in a controlled-flow liver model. Med. Phys..

[7] Li Z.X., Kawashita M., Araki N., Mitsumori M., Hiraoka M., Doi M. (2010). Magnetic SiO_2_ gel microspheres for arterial embolization hyperthermia. Biomed. Mater..

[8] Adedoyin A.A., Ekenseair A.K. (2018). Biomedical applications of magneto-responsive scaffolds. Nano Res..

[9] Tartaj P., Morales M.D., Veintemillas-Verdaguer S., Gonzalez-Carreno T., Serna C.J. (2003). The preparation of magnetic nanoparticles for applications in biomedicine. J. Phys. D Appl. Phys..

[10] Jozefczak A., Hornowski T., Rozynek Z., Skumiel A., Fossum J.O. (2013). Rheological study of dextran-modified magnetite nanoparticle water suspension. Int. J. Thermophys..

[11] Cvek M., Mrlik M., Ilcikova M., Mosnacek J., Babayan V., Kucekova Z., Humpolicek P., Pavlinek V. (2015). The chemical stability and cytotoxicity of carbonyl iron particles grafted with poly(glycidyl methacrylate) and the magnetorheological activity of their suspensions. RSC Adv..

[12] Vasiliev V.G., Sheremetyeva N.A., Buzin M.I., Turenko D.V., Papkov V.S., Klepikov I.A., Razumovskaya I.V., Muzafarov A.M., Kramarenko E.Y. (2016). Magnetorheological fluids based on a hyperbranched polycarbosilane matrix and iron microparticles. Smart Mater. Struct..

[13] Fu Y., Yao J.J., Zhao H.H., Zhao G., Wan Z.S., Qiu Y. (2018). Bidisperse magnetic particles coated with gelatin and graphite oxide: Magnetorheology, dispersion stability, and the nanoparticle-enhancing effect. Nanomaterials.

[14] Parikh N., Parekh K. (2015). Technique to optimize magnetic response of gelatin coated magnetic nanoparticles. J. Mater. Sci. Mater. Med..

[15] Intorasoot S., Techateerawat J., Intorasoot A. (2013). Genomic DNA isolation from dried blood using gelatin-coated magnetic particles. Curr. Sci..

[16] Intorasoot S., Srirung R., Intorasoot A., Ngamratanapaiboon S. (2009). Application of gelatin-coated magnetic particles for isolation of genomic DNA from bacterial cells. Anal. Biochem..

[17] Wang G.S., Ma Y.Y., Tong Y., Dong X.F. (2017). Development of manganese ferrite/graphene oxide nanocomposites for magnetorheological fluid with enhanced sedimentation stability. J. Ind. Eng. Chem..

[18] Rendos A., Li R., Woodman S., Ling X., Brown K.A. (2021). Reinforcing magnetorheological fluids with highly anisotropic 2d materials. ChemPhysChem.

[19] Jamari S.K.M., Nordin N.A., Ubaidillah Aziz S.A.A., Nazmi N., Mazlan S.A. (2020). Systematic Review on the Effects, Roles and Methods of Magnetic Particle Coatings in Magnetorheological Materials. Materials.

[20] De Vicente J., Klingenberg D.J., Hidalgo-Alvarez R. (2011). Magnetorheological fluids: A review. Soft Matter.

[21] Zhang J.T., Song W.L., Peng Z., Gao J.W., Wang N., Choi S.B., Kim G.W. (2020). Microstructure simulation and constitutive modelling of magnetorheological fluids based on the hexagonal close-packed structure. Materials.

[22] Fu Y., Yao J.J., Zhao H.H., Zhao G., Wan Z.S., Qiu Y. (2018). Fabrication and magnetorheology of bidisperse magnetic microspheres coated with gelatin and multi-walled carbon nanotubes. Smart Mater. Struct..

[23] Belyavskii S.G., Mingalyov P.G., Giulieri F., Combarrieau R., Lisichkin G.V. (2006). Chemical modification of the surface of a carbonyl iron powder. Protect. Met..

[24] Cvek M., Mrlik M., Ilcikova M., Plachy T., Sedlacik M., Mosnacek J., Pavlinek V. (2015). A facile controllable coating of carbonyl iron particles with poly(glycidyl methacrylate): A tool for adjusting MR response and stability properties. J. Mater. Chem. C.

[25] Xiao S.J., Textor M., Spencer N.D., Sigrist H. (1998). Covalent attachment of cell-adhesive, (Arg-Gly-Asp)-containing peptides to titanium surfaces. Langmuir.

[26] Saw M.M., Chandler B., Ho K.M. (2012). Benefits and risks of using gelatin solution as a plasma expander for perioperative and critically ill patients: A meta-analysis. Anaesth. Intensive Care.

[27] Phillips G.O., Williams P.A. (2009). Handbook of Hydrocolloids.

[28] Gaihre B., Khil M.S., Lee D.R., Kim H.Y. (2009). Gelatin-coated magnetic iron oxide nanoparticles as carrier system: Drug loading and in vitro drug release study. Int. J. Pharm..

[29] Chang M.C., Tanaka J. (2002). FT-IR study for hydroxyapatite/collagen nanocomposite cross-linked by glutaraldehyde. Biomaterials.

[30] Machovsky M., Mrlik M., Kuritka I., Pavlinek V., Babayan V. (2014). Novel synthesis of core-shell urchin-like ZnO coated carbonyl iron microparticles and their magnetorheological activity. RSC Adv..

[31] Mrlik M., Sedlacik M., Pavlinek V., Bazant P., Saha P., Peer P., Filip P. (2013). Synthesis and magnetorheological characteristics of ribbon-like, polypyrrole-coated carbonyl iron suspensions under oscillatory shear. J. Appl. Polym. Sci..

[32] Konig R., Muller S., Dinnebier R.E., Hinrichsen B., Muller P., Ribbens A., Hwang J., Liebscher R., Etter M., Pistidda C. (2017). The crystal structures of carbonyl iron powde—Revised using in situ synchrotron XRPD. Z. Krist. Cryst. Mater..

[33] Plachy T., Cvek M., Munster L., Hanulikova B., Suly P., Vesel A., Qilin C. (2021). Enhanced magnetorheological effect of suspensions based on carbonyl iron particles coated with poly(amidoamine) dendrons. Rheol. Acta.

[34] Cvek M., Mrlik M., Pavlinek V. (2016). A rheological evaluation of steady shear magnetorheological flow behavior using three-parameter viscoplastic models. J. Rheol..

[35] Plachy T., Kutalkova E., Sedlacik M., Vesel A., Masar M., Kuritka I. (2018). Impact of corrosion process of carbonyl iron particles on magnetorheological behavior of their suspensions. J. Ind. Eng. Chem..

[36] Ginder J.M., Davis L.C. (1994). Shear stresses in magnetorheological fluids—Role of magnetic saturation. Appl. Phys. Lett..

[37] De Sousa S.R.G., dos Santos M.P., Bombard A.J.F. (2019). Magnetorheological gel based on mineral oil and polystyrene-b-poly(ethene-co-butadiene)-b-polystyrene. Smart Mater. Struct..

[38] Marins J.A., Plachy T., Kuzhir P. (2019). Iron-sepiolite magnetorheological fluids with improved performances. J. Rheol..

